# Phenomenologic analysis of healthcare worker perceptions of intensive care unit diaries

**DOI:** 10.1186/cc11938

**Published:** 2013-01-21

**Authors:** Antoine Perier, Anne Revah-Levy, Cédric Bruel, Nathalie Cousin, Stéphanie Angeli, Sandie Brochon, François Philippart, Adeline Max, Charles Gregoire, Benoit Misset, Maité Garrouste-Orgeas

**Affiliations:** 1 Maison des Adolescents, University Hospital Cochin; 75014 Paris, France; 2 INSERM U-669, Paris Sud University and Paris Descartes University, UMR-S0669, Paris, France; 3 Centre de Soins Psychothérapiques pour Adolescents, Argenteuil Hospital, Argenteuil, France; 4 Medical ICU, Saint Joseph Hospital Network, 75014 Paris, France; 5 René Descartes University, 75005 Paris, France; 6 University Joseph Fourier, Integrated Research Center U823 "Epidemiology of Cancers and Severe Diseases," Albert Bonniot Institute, Rond-point de la Chantourne, 38706 La Tronche Cedex, France

## Abstract

**Introduction:**

Studies have reported associations between diaries kept for intensive care unit (ICU) patients and long-term quality-of-life and psychological outcomes in patients and their relatives. Little was known about perceptions of healthcare workers reading and writing in the diaries. We investigated healthcare worker perceptions the better to understand their opinions and responses to reading and writing in the diaries.

**Methods:**

We used a phenomenologic approach to conduct a qualitative study of 36 semistructured interviews in a medical-surgical ICU in a 460-bed tertiary hospital.

**Results:**

Two domains of perception were assessed: reading and writing in the diaries. These two domains led to four main themes in the ICU workers' perceptions: suffering of the families; using the diary as a source of information for families but also as generating difficulties in writing bad news; determining the optimal interpersonal distance with the patient and relatives; and using the diary as a tool for constructing a narrative of the patient's ICU stay.

**Conclusions:**

The ICU workers thought that the diary was beneficial in communicating the suffering of families while providing comfort and helping to build the patient's ICU narrative. They reported strong emotions related to the diaries and a perception of intruding into the patients' and families' privacy when reading the diaries. Fear of strong emotional investment may adversely affect the ability of ICU workers to perform their duties optimally. ICU workers are in favor of ICU diaries, but activation by the diaries of emotions among younger ICU workers may require specific support.

## Introduction

Patient diaries were first used in Denmark, Sweden, and Norway, and then introduced several decades later in other European countries, such as the UK, Switzerland, and, finally, France [1]. Diary entries were found to fall into four main categories: sharing the story, sharing the presence, sharing feelings, and sharing through support [2]. The multiple roles of diaries kept for ICU patients include reconstruction of the illness narrative [3], communication of caring intent [4], debriefing to deal with posttraumatic stress syndrome (PTSD) [5], and the promotion of family healing [3]. ICU diaries have been reported to help in the transition of patients from critical illness to normalcy [2,5-8].

We previously evaluated the effect of an ICU diary on the well-being of patients and families and found that the diary decreased posttraumatic stress-related symptoms in both at 1 year after ICU discharge [1]. Our diaries, written in everyday language, included entries by ICU workers (including physicians) and relatives that were designed to help the patients to understand what happened to them during the ICU stay. Of 5,208 sentences in the 59 diaries used for the present study, 17.4% were written by ICU physicians, 22.7% by nurses and nursing assistants, and 59.8% by relatives [1].

Few data exist about the opinions of ICU workers regarding diaries. A qualitative study of ICU nurses in Sweden showed that the diary was perceived as giving the patient ownership of the ICU experience and recapitulating the ICU stay, although time constraints were cited as a reason to not open a diary [9]. The nurses thought that reading the diaries helped to guide patient care but also reported negative effects, such as fear of reading what was in the diary [9]. Similarly, a study consisting of interviews of one nurse in each ICU in Denmark identified negative perceptions, such as feelings that experience was required to write in diaries for unconscious patients and that the diary constituted additional paperwork [10]. The objective of this study was to assess the perceptions of ICU workers contributing to the diaries.

## Materials and methods

### Setting and participants

The ICU has 10 single-bed rooms and admits about 400 patients each year. Family members can visit around the clock, stay as long as they wish, and participate in patient care with the nurses. The ICU has a patient/nurse ratio of 2.5. The day team includes five full-time physicians and four residents.

The criterion for including ICU workers in the study was experience with ICU diaries. To develop a comprehensive understanding of our study question, we used a sample of ICU healthcare workers who acquired experience with ICU diaries between May 2008 and November 2009, the earliest period of diary use in our ICU. In each category of participants (physicians, nurses, and nursing assistants), new participants were included until data saturation occurred.

### Collection and analysis of data

In December 2009, we used semistructured in-depth interviews to assess ICU workers' perceptions of the 59 diaries kept in our ICU. The characteristics of the patients for whom diaries were established are described elsewhere [1]. The interview was performed in the ICU in a quiet place by using an interview guide (Table 1) built by three physicians (MGO, CB, FP) and three ICU nurses (NC, SA, SB). All interviews were performed by the same physician (MGO), audio-recorded with the interviewees' permission, transcribed verbatim, and evaluated by using interpretative phenomenologic analysis [11]. First, the transcripts were checked for accuracy against the audio recordings. Each transcript was read several times and then coded to identify initial themes, which were noted in the margins. This procedure closely resembled free textual analysis. Each reading had the potential to generate new insights. Then, themes recurring across transcripts were identified; recurring themes reflect a shared understanding of the phenomenon in question among participants. This stage involved a more analytic ordering of the data, as the researchers tried to make sense of the connections linking themes. Some of the themes tended to cluster. The process was dynamic and cyclic, with each transcript leading to the collection of further data and to their subsequent analysis. The aim was to recognize ways in which narratives from the participants were similar but also different. The researchers strove both to identify recurring patterns and to detect new issues, to take into account convergences and divergences in the data. The last stage consisted of producing a coherent ordered list of the themes.

**Table 1:** Interview guide

Question 1	What reflections and feelings did you have when you read the diaries?
Question 2	How did the diaries modify your relationship with the patients or families?

Question 3	How did the diaries modify the information you delivered to the families?

Question 4	How did you consider writing in the diaries to be a burden on your working schedule?

Question 5	How did you consider the diaries useful for the patients or families?

A clinical psychologist (AP) carried out the main analysis, and physicians (MGO, ARL) read the transcripts to improve the consistency and coherence of the analysis by ensuring that identified themes accurately reflected the data and that the analysis was not confined to a single perspective. The results were discussed during multiple meetings of the entire study team to determine when clarification was in order and whether the themes should be modified. To increase internal validity further, we distinguished clearly between statements by the interviewees and interpretations or accounts of those statements by the researchers [11,12]. In the Results section, we supply examples of interviewees' statements to illustrate recurring themes. To protect confidentiality, identifying information was deleted from the statements. For this report, the statements were translated from French to English, taking care to preserve their meaning and feeling tone.

### Ethical committee approval

The study was approved by the ethics committee (Comité de Protection des Personnes) of the Pitié-Salpêtrière Hospital, which waived the requirement for written informed consent. ICU workers participated on a voluntary basis and gave their permission for audio recording of their interviews. For each interview, the job title was indicated.

## Results

The participant characteristics are displayed in Table 2. Interpretive phenomenologic analysis identified two domains of experience: writing in and reading the diaries. Four themes were identified: suffering of the families, giving information to families, determining the optimal interpersonal distance with patients and families, and reconstructing the patient's story.

**Table 2:** Characteristics of the ICU staff members

Variables	Nurses *n *= 23	Nursing assistants *n *= 4	Physicians *n *= 9
Age, years	30.5 ± 4.3	33.7 ± 3.1	36.4 ± 8.1

Females, number (%)	21 (91.3)	3 (75)	5 (50)

Senior physicians, number (%)			5 (50)

ICU experience, years, mean ± SD	3.5 ± 2.5	4.6 ± 2.2	9.1 ± 8.3

ICU, intensive care unit; SD, standard deviation.

### The suffering of families

The ICU workers focused on the overwhelming emotions experienced by families in relation to the ICU stay and on the manner in which families coped with these emotions. Writing in the diary was considered useful to the families as a means of communicating their emotions. The opportunity to vent emotions was viewed as beneficial in helping the families cope with the anxiety generated by the ICU experience.

"The diary is a valuable emotional outlet for the families, who can describe their feelings and experience in writing (Nurse 7)." "I feel that the families unload their stress onto the diary, and sometimes it's huge!" (Nurse 10). "It's an outlet for the family, it releases all the anxiety while setting limits; you can't say everything either!"(Nurse 4). "The diary allows the family to express their feelings, and that helps to relieve the anxiety" (Physician 5).

### Giving information to the families

The ICU staff members thought it was important that they write in the diaries. They believed that writing in the diaries helped to improve communication with the families and served as an adjunct to the information delivered orally.

"The diary serves as a link between the family and the staff. It helps to communicate information. And it leads the family to think about what's happening and then to talk about it with us, about what is going on every day and not only about the prognosis" (Resident 1).

"The diary doesn't change the information given orally to the patients but it allows us to deliver this information in a different way, according to what we feel is right for that family" (Nurse 14). "The diary serves as a complement to the oral information given during the stay" (Nurse 11).

The diary was perceived as creating a useful record that gave families enough time to assimilate the information, in a setting in which the staff had to cope with a shortage of time. "The diary changes the impact of the information supplied to families, because it is a written record that they can read as often as they want, whereas oral information may be incompletely heard or understood by people who are under stress" (Physician 1).

"It improves the transparency of the information that we give to the patient and family, and the information is clearer, because we take the time to choose the best words" (Physician 2).

Conversely, the ICU workers were concerned about the handling of negative events and bad news. This concern was related to a feeling of having failed patients whose clinical condition deteriorated and to worries about the impact of bad news on the family.

"I don't write when the patients are not doing well; it's too hard to face the feeling of failure" (Nurse 9). "It's hard to write when I don't see any hope for life after the ICU" *(Nurse 4)*. "Should we write about serious problems? How can we handle the fact that the family will read it?" (Resident 3).

The perceived impact on the families of reading the diaries differed with the experience of the ICU workers. Experienced ICU workers thought that reading the diaries was a source of considerable support and encouragement to the families, not only through the information written by the ICU staff, most notably about signs of progress, but also through the descriptions of everyday care procedures.

"When the patient does well, I feel encouraged" (Nurse 17). "It provides psychological support to the families" (Nurse 13)."I don't know whether it's therapeutic, but we can communicate information, and writing down what we said earlier helps the family"(Nurse 9).

ICU workers with limited experience, in contrast, emphasized the anxiety-generating potential of the diary information.

### Interpersonal distance with families and patients

#### Humanization

Writing in the diary was perceived by the ICU workers as helping them to humanize their roles by emphasizing the dimension of sensitivity and empathy in their interactions with the patient and family.

"The diary also shows the family that we care about their loved one, although they see many of our interventions as acts of "violence" (Nurse 15). "Writing helps us to remain human, because in the ICU, we tend to keep a lot of distance" (Nurse 4). "It's important to be able to show the family that we truly care about the patient" (Physician 1).

The ICU workers emphasized the importance of having their diary entries read by the family. They thought that the diary helped them to cast their work in a more human light and testified to their commitment to the patient.

"A means for the family of putting names and personalities to the staff members, with whom they have limited oral communication; it improves the interactions" (Nursing assistant 1)."The families realize that we are committed, and that makes us more human"(Nurse 6). "It makes things a bit more human for the families" (Physician 3).

The ICU workers also read the diaries. They thought that the diary entries by the family broadened their perception of the family to encompass a more-sensitive and human dimension, by revealing the family's worries and distress, as well as their commitment to and feelings for the patient.

"It makes us realize that the ICU is rough on families; for us, the staff, we get used to it (Physician 4). "Sometimes, reading the diaries gives us a feeling of compassion; we are moved by the accounts"(Nurse 10)."It adds a human dimension by revealing what the families go through, because as healthcare workers, we tend to maintain a lot of distance" (Resident 3). "When the families write loving words, it's beautiful, it's moving, and that's a good thing for us technical people" (Nurse 12).

Another important benefit from the diary, according to the ICU workers, was that it would be read later on by the patient, who would then see that he or she was cared for as a human being and not as a physical object.

"The diary allows us to say things to the patients, and when they read our entries, it's important that they realize that we cared about them as people" (Nurse 7). "It's a good thing that the patient can realize that we provided care in a sensitive way and that we worried, because sometimes the interventions were distressing" (Nursing assistant 3). "It's important that the patient be aware that we consider him or her as a human being" (Nurse 11).

#### Space for emotions

Although the ICU workers thought that it was important to give a human dimension to their work, they also perceived affects and emotions as problematic, as emotional involvement was viewed as potentially distressing for the ICU workers and, therefore, as carrying a risk of suboptimal professional performance.

"It's complicated; I can't manage to write everyday things, and its hard to write about what I feel...I'm not a member of their family; I'm a nurse" (Nurse 13). "I can't write subjective things.... As healthcare workers, we have to anesthetize our emotions, we can't let ourselves become too emotional, because if we did, we would be overwhelmed" (Nurse 4). "I believe that for the healthcare workers, it's important to stick with the technical issues, the facts" (Physician 3).

Reading the diaries also highlighted the difficulties experienced by ICU workers in finding what might be the optimal interpersonal distance with the patients and families.

"Too close to the patients and families, and then we can't do our job; too great an emotional distance, and that's difficult, too" (Resident 4). "The diary makes us feel a bit closer to the patients, but I don't know whether that's good or bad" (Nurse 8). "We're not just technicians; it's important to be close to the patients and families, but then we are vulnerable" (Nurse 2).

#### Intruding into the family's intimacy

A perception that emerged with considerable force from the interviews was that of intruding into the intimacy of the families. Reading the families' entries was thought to violate the patients' and families' privacy.

"The families write about very personal things. When I read the diaries, I almost feel as if I am intruding" (Resident 4). "So I felt very uncomfortable; I felt it was very personal; yes, really uncomfortable, although this intimate contact was "allowed," because the families know we will read their entries; it makes me uncomfortable" (Nurse 16). "When I read the diaries, it's very powerful; I feel that I am entering into the patients' intimacy; it makes a very strong impression" (Resident 1)."When I read the diary, I felt as if I were becoming part of the family, so I quickly closed the diary; I ducked back behind the wall" (Resident 4).

#### Reconstruction of the patient's story

When asked whether reading the diary was useful to the patients, the main perception was that the diary served as a record of what happened in the ICU. The ICU workers thought that the diary both provided a description of medical events as they occurred over time and gave an account of life events (for example, pertaining to the family, society, and culture) as they unfolded around the patient.

"There is enough there to allow the patient to remember or to imagine what happened in the ICU" (Physician 1)."The diary is important to help the patient remember" (Nursing assistant 2). "The diary has a therapeutic role for the patient: many patients come back to see us because they want to know what happened to them" (Physician 4).

## Discussion

This study explored the perceptions of diaries by ICU workers. The ICU workers thought that the diaries helped the relatives to cope with their anxiety and other strong emotions, provided information to the relatives, provided a narrative of the patient's stay, and contributed to humanizing the ICU. They also expressed two difficulties when reading the diaries: strong emotions and a feeling of violating the privacy of the patients and families.

The ICU workers thought that writing in the diaries helped the relatives to express their strong emotions, thereby decreasing the level of anxiety and contributing to regulate stress. ICU admission of a loved one has been shown to induce stress, with symptoms of anxiety or depression in 75.5% of family members overall and 82.7% of spouses [13]. The ICU stay was often followed by quality-of-life impairments in the patients and relatives, among whom a substantial proportion exhibited symptoms of posttraumatic stress [14]. The diary was perceived in our study as a link between the patient, relatives, and ICU team.

However, the diary was not associated with greater satisfaction or decreased symptoms of anxiety at ICU discharge in our previous study [1], contrary to the perceptions of the ICU workers in the present study. Interviews of 13 relatives of ICU patients 6 to 12 months after ICU discharge indicated that the diaries participated in their healing [3]. The complex relation between relatives and diaries will be the subject of our next qualitative evaluation.

The ICU workers believed the diaries were a useful source of information for the relatives. Clear and honest information is among the well-established needs of families of ICU patients. Families want their questions answered honestly, using words they can understand easily [15]. French multicenter studies showed that the first meeting with the relatives lasted only 15 minutes and that half the relatives did not understand the diagnosis, prognosis, or treatment of their loved ones [16,17]. Use of written information in addition to oral information may improve comprehension among families. For instance, a leaflet providing general ICU information, including the names of the devices used in the ICU, improved comprehension [18]. The diary is of particular interest as a means of supplying specific information about the patient, thus supporting the oral information delivered by the physician. We found differences in perceptions between senior physicians and residents. Senior physicians were confident they could supply specific information in the diaries, whereas residents were concerned that providing distressing news in writing might be deleterious. This point is important to consider in ICUs that plan to start using diaries. Our previous study describing the content of the ideal diary may help to train younger physicians [1].

An important strength of the diary, as perceived by the ICU workers, was that it humanized the ICU and supplied personal information on the patients and relatives. The diary was perceived as evidence of a caring and commited relationship of the ICU workers with the patient. Thus, despite the highly technical nature of the interventions in the ICU, the patient would be able to understand that he or she was perceived as a human being. The feelings and representations described by the ICU workers in relation to writing in and reading the diaries also emphasized humanization of the families. The ICU workers were able to read about the families' pain and anxiety, their affection and tenderness for the patient, and sometimes their sympathy and gratitude toward the ICU workers. These emotions expressed by the families in the diaries sometimes generated strong feelings in the ICU workers. This finding may be related to the participation of the entire ICU team in keeping the diaries in our unit and to the study having been done shortly after diaries were introduced in our unit, which may have contributed to the strength of the emotions experienced by the ICU workers. Some of the ICU workers expressed feelings that constituted defense mechanisms against emotional involvement. Thus, our findings highlight the tension between being close to the families to support them better and maintaining the emotional distance needed to ensure optimal professional performance. Several studies have emphasized the therapeutic impact of empathy, emotional intelligence, and emotional labor of ICU workers [19-22]. The difficulty in establishing a balance between pressure at work, including that related to highly emotional situations, and the need for developing coping strategies has been pointed out by ICU workers [23]. Similarly, some of the ICU workers interviewed in our study thought that reading about the patients and families in the diaries was distressingly intrusive. Although the diaries were intended to be read by all those who wrote in them, the personal nature of some of the entries made the ICU workers feel as if they were entering into private territory. This concern about being unduly intrusive led to anxiety and to a number of defense mechanisms. However, several studies have established the importance of identifying and regulating emotions [19,24,25].

"Filling the memory gap" by constructing the narrative of the illness has been a major reason for using ICU diaries [26]. The ICU workers in our study agreed that this was an important function of the diaries. The diaries were perceived as supplying material that would help the patient distinguish delusional memories from real events [27]. Fear, sleep deprivation, and delirium are well-known complications of the ICU stay. Delirium has been reported in 34.2% to 84% of ICU patients [28-30] and independently influenced the duration of mechanical ventilation and the length of the ICU stay [31]. Risk factors for ICU delirium were investigated recently [30]. Delirium contributes to the loss of recollections about events in the ICU and to the development of delusional memories, which are associated with depression [32] and posttraumatic stress-related symptoms [33-35]. The day-by-day description of events in the diary by the ICU workers and relatives is valuable to patients seeking to understand what happened to them in the ICU and thereby to regain a feeling of ownership of their own lives. The diary not only served as an additional source of information, but also improved the relationship between relatives and ICU workers and, later, between patients and ICU workers. The diary may be useful in the rehabilitation process after ICU discharge [36].

This study has strengths and limitations. Individual interviews allowed us to obtain in-depth insights into the different reported factors and their interactions but did not provide a broad picture. The interview method does not allow the sharing and comparison of perceptions that occurs during the interactions typical of focus groups. Our data may not be generalizable to ICUs that have long-term experience with ICU diaries, as our study was conducted after 6 months of experience of diaries in our ICU.

## Conclusions

In conclusion, this study describes the experience of healthcare workers of an ICU about the use of diaries. Our participants perceived the diaries as beneficial for the patients and families but also described them as a source of strong emotions and as possibly violating the privacy of patients and families. This study provides new insights. On a practical level, apart from the logistical issues raised by the use of diaries, the role for emotions activated by the diaries and having a potential for influencing relationships of ICU workers with patients and families deserves careful attention. In particular, younger ICU workers may need to receive specific support.

## Key messages

• The healthcare workers perceived the diaries as beneficial for the patients and their families.

• The diaries provided comfort and helped in building the patient's ICU narrative.

• The diaries humanized the ICU and supplied personal information on the patients and relatives.

• The healthcare workers emphasized the strong emotions activated by reading in the diaries and expressed difficulties in determining what might be the optimal interpersonal distance with the patients and families.

• This study adds new insights into the implementation of diaries in ICUs.

## Abbreviations

ICU: intensive care unit; PTSD: posttraumatic stress disorder.

## Competing interests

The authors declare that they have no competing interests.

## Authors' contributions

ARL, AP, MGO, CB, FP, NC, SB, and SA conceived and designed the study. MGO acquired the study data. ARL, AP, and MGO analyzed and interpreted the study data. ARL, AP, and MGO wrote the manuscript. FP, CG, AM, and BM contributed to the final revision of the manuscript for important intellectuel content. All the authors read and approved the final manuscript.
